# Défi diagnostique et thérapeutique de la neuromyélite optique en Côte d’Ivoire : à propos d’un cas pédiatrique

**DOI:** 10.48327/mtsi.v6i1.2026.707

**Published:** 2026-01-13

**Authors:** Claude Valéry Cédric Aka KADJO, Constance YAPO-EHOUNOUD, Reine Prisca AGBOHOUN, Arlette Désirée AKA

**Affiliations:** 1 Université Félix Houphouët-Boigny, UFR Sciences médicales Abidjan, service de neurologie médicale, Côte d’Ivoire; 2 Université Félix Houphouët-Boigny, UFR Sciences médicales Abidjan, service d’ophtalmologie, Côte d’Ivoire

**Keywords:** Neuromyélite optique, Enfant, Diagnostic, Traitement, Côte d’Ivoire, Afrique subsaharienne, Optic neuromyelitis, Child, Diagnosis, Treatment, Côte d’Ivoire, Sub-Saharan Africa

## Abstract

**Introduction:**

La neuromyélite optique (NMO) est une pathologie inflammatoire, démyélinisante du système nerveux central (SNC) évoluant généralement par poussées. L’âge moyen de début se situe vers 40 ans, mais il existe des formes pédiatriques. Nous décrivons notre premier cas pédiatrique, et en discutons les particularités.

**Observation clinique:**

L’enfant âgée de 8 ans, est hospitalisée en juin 2023, pour déficit moteur des quatre membres. En novembre 2021, elle avait présenté une baisse binoculaire de l’acuité visuelle suivie d’une récupération partielle puis, 11 mois après, un second épisode plus sévère sans récupération. En mai 2023, elle présente des vomissements incoercibles associés à un déficit moteur discret des quatre membres. En juin 2023, elle décrit une asthénie importante, un prurit facial marqué, et une aggravation du déficit moteur. L’EDSS (*Expanded Disabililty Status Scale*) a été estimé à 8. Sur le plan ophtalmologique on note une absence de perception lumineuse et une atrophie optique aux fonds d’œil. L’exploration permet de confirmer une NMO séropositive. Après un bolus de corticothérapie, on note une amélioration clinique avec un EDSS à 5,5. Après rechute sous aziathioprine, l’évolution est restée stable sous rituximab.

**Discussion:**

La NMO de l’enfant représente 3 à 5 % de l’ensemble des NMO et est souvent sous-diagnostiquée. On note une hétérogénéité clinico-radiologique dans les NMO pédiatriques. Les formes trompeuses telles que le syndrome de l’area prostema, assez fréquentes chez l’enfant, ne doivent pas être méconnues. Les traitements de fond reposent sur les immunosuppresseurs.

**Conclusion:**

La NMO est une affection rare et méconnue en Afrique subsaharienne dont les poussées représentent de véritables urgences thérapeutiques.

## Abstract (en)

**Introduction:**

Neuromyelitis optica (NMO) is an inflammatory and demyelinating disease of the central nervous system (CNS) that typically progresses in episodes. The average age of onset is around 40 years old, though there are also pediatric forms. We present our first pediatric case and discuss its unique features.

**Clinical observation:**

An 8-year-old child was hospitalized in June 2023 due to motor deficits in all four limbs. In November 2021, she presented with a binocular decrease in visual acuity, followed by partial recovery. Eleven months later, she experienced a second, more severe episode without recovery. In May 2023, she presented with uncontrollable vomiting and a mild motor deficit in all four limbs. By June 2023, she reported significant asthenia, severe facial itching, and worsening motor deficits. Her EDSS score was estimated at 8. Ophthalmologically, she had an absence of light perception and optic atrophy on fundus examination. Testing confirmed seropositive NMO. Following corticosteroid therapy, she showed clinical improvement with an EDSS score of 5.5. Following a relapse on azathioprine, her condition remained stable on rituximab.

**Discussion:**

NMO accounts for 3 to 5% of all NMO cases in children and is often underdiagnosed. There is clinical and radiological heterogeneity in pediatric NMO. Misleading forms, such as area prostema syndrome (which is quite common in children), should not be overlooked. Basic treatments are based on immunosuppressants.

**Conclusion:**

NMO is a rare and little-known condition in sub-Saharan Africa, with flare-ups representing genuine therapeutic emergencies.

## Introduction

La neuromyélite optique (NMO) est une pathologie inflammatoire, démyélinisante du système nerveux central (SNC) évoluant généralement par poussées. Longtemps considérée comme une variante de la sclérose en plaques (SEP), elle correspond en fait à une physiopathologie, une symptomatologie et une prise en charge différentes de celles de la SEP [11]. Les derniers critères diagnostiques de Wingerchuk de 2015 [10] permettent de mieux en faire le diagnostic. La découverte de l’anticorps anti aquaporine 4 a contribué à élargir de manière considérable les phénotypes cliniques associés à la NMO, aboutissant au concept de « spectre de la NMO ». Les troubles du spectre de la NMO (*Neuromyelitis Optica Spectrum Disorders*, NMOSD) affectent beaucoup plus fréquemment la femme que l’homme (7 pour 1 chez l’adulte; 3 pour 1 chez l’enfant). L’âge moyen de début se situe vers 40 ans, mais il existe des formes pédiatriques et des formes à début très tardif (après 80 ans) [4]. La NMO est peu connue et sous diagnostiquée en Afrique subsaharienne où les maladies infectieuses sont connues comme causes importantes de myélopathie [6]. La forme de l’enfant est très peu étudiée en Afrique. Nous décrivons notre premier cas pédiatrique, et en discutons les particularités cliniques, diagnostiques, thérapeutiques et évolutives.

## Observation clinique

Une enfant âgée de 8 ans, ivoirienne, est admise aux urgences de pédiatrie le 15 juin 2023 pour déficit moteur des quatre membres associé à une asthénie marquée. L’interrogatoire retrouve un premier épisode de baisse binoculaire de l’acuité visuelle en novembre 2021. Aucune étiologie n’avait été retenue et l’évolution a été marquée par une amélioration incomplète de l’acuité visuelle. En septembre 2022, la patiente présente un second épisode de baisse binoculaire de l’acuité visuelle (BAV) sévère sans récupération secondaire. Les données de l’examen ophtalmologique n’ont pas été précisées. L’IRM encéphalique réalisée en septembre 2022 n’avait retrouvé aucune lésion parenchymateuse.

En mai 2023, elle a été admise aux urgences médicales pour des vomissements incoercibles associés à un déficit moteur discret des quatre membres. L’exploration par fibroscopie oeso-gastro-duodénale a été non contributive quant à la recherche étiologique. Cependant l’évolution après prise en charge à visée digestive a été marquée par un amendement des vomissements et une persistance du déficit moteur.

**Figure 1:**
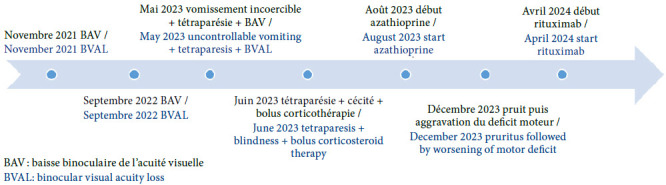
Résumé chronologique des poussées cliniques et du traitement

L’histoire récente (Fig. 1) note, 3 jours avant son admission aux urgences, une asthénie importante associée à un prurit facial marqué, des paresthésies diffuses et une aggravation du déficit moteur. L’examen neurologique à son admission a mis en évidence : un syndrome tétra pyramidal spastique avec une force motrice cotée à 2/5, une dysesthésie diffuse, une dysphonie et une cécité binoculaire avec mydriase aréactive. L’examen en ophtalmologie n’a pas été réalisé. L’échelle de cotation du handicap, *Expanded Disability Status Scale* (EDSS) était estimée à 8. Devant la spécificité connue des auto-anticorps, au vu de la présentation clinique et des limites financières de la famille, la priorité des explorations a été en faveur des explorations biologiques. L’hypothèse d’une pathologie inflammatoire a été évoquée et confirmée par la mise en évidence dans le sérum d’anticorps (Ac) anti-aquaporine 4 (AQP4). Les anticorps *anti-myelin oligodendrocyte glycoprotein* (MOG) étaient négatifs. Aucune imagerie du SNC n’a été réalisée au décours de cet épisode en raison des limites financières de la famille. La patiente a bénéficié de bolus de corticoïdes à raison de 30 mg/kg/jr pendant 5 jours puis un relais à 1 mg/kg/ jr à dose régressive pendant 1 mois. L’évaluation en fin de corticothérapie a noté un score EDSS à 5,5 et sur le plan ophtalmologique une absence de perception lumineuse, une semi-mydriase aréflexique et une atrophie optique au fond d’œil. Du fait des difficultés d’accès aux traitements anti-CD20, un traitement immunosuppresseur par azathioprine a été proposé comme traitement de fond. L’évolution sous traitement a été marquée par une rechute avec aggravation du déficit moteur quatre mois après le début de l’azathioprine. Nous avons préconisé une escalade thérapeutique marquée par l’introduction du rituximab obtenu par l’intermédiaire d’un circuit de gratuité pour les patients NMOSD AQP4+, courant avril 2024. La patiente est restée stable sous rituximab (protocole 500 mg à J0 puis 500 mg à J15 et 1g tous les 6 mois) avec un score EDSS à 5.

## Discussion

En Côte d’Ivoire, comme dans d’autres pays tropicaux à ressources limitées, la priorité diagnostique reste orientée vers les maladies infectieuses (paludisme, tuberculose, VIH), reléguant souvent les pathologies inflammatoires démyélinisantes au second plan. Ces pathologies inflammatoires sont de diagnostic et de prise en charge difficiles. Le coût de la prise en charge par rituximab, actuellement utilisé en première ligne dans le traitement de fond de la NMO (coût ≈584 348 FCFA soit 890 €) reste essentiellement à la charge de la famille, ce qui demeure un facteur limitant essentiel dans notre contexte de travail. L’accès au rituximab a été facilité à partir d’avril 2024 grâce à un programme de gratuité instituée au CHU de Cocody (Abidjan) pour les patients NMOSD AQP4+, mais les critères d’éligibilité, la pérennité et la couverture géographique de ce programme demeurent incertains. Ce contexte met en lumière la nécessité d’une politique de santé publique proactive en matière de maladies rares neurologiques.

La NMO est une maladie inflammatoire du SNC qui affecte principalement les nerfs optiques et la moelle épinière [12]. La NMO est plus fréquente chez les personnes d’origine asiatique et africaine que chez celles d’origine européenne, cependant il existe peu de données sur la NMO en Afrique [6]. Les étiologies des myélites selon les données en Afrique subsaharienne sont dominées par les maladies infectieuses, notamment la tuberculose, suivies des causes néoplasiques [2]. La plupart des études en Afrique subsaharienne sur la NMO ont noté un retard diagnostique [6], qui pourrait être lié à la méconnaissance de cette affection.

La NMO pédiatrique est moins fréquente que celle de l’adulte. Les données actuelles suggèrent que la NMO pédiatrique représente 3 à 5 % de tous les cas de NMO, selon les critères diagnostiques et l’inclusion du test d’anticorps AQP4 [9]. L’incidence et la prévalence de la NMO pédiatrique ne sont pas bien caractérisées [7]. À notre connaissance, il n’existe pas de données épidémiologiques de population en Afrique subsaharienne de la NMO chez l’enfant. Nous en décrivons notre premier cas confirmé.

Dans une vaste étude multicentrique menée par la Mayo Clinic [5] chez des patients pédiatriques atteints de NMOS, l’âge médian au début des symptômes était de 12 ans (intervalle de 4 à 18), avec une prépondérance féminine claire (88 %). Nous présentons un cas d’une enfant ivoirienne particulièrement jeune. Une étude sur un plus grand nombre de cas permettrait de préciser l’âge moyen de survenue de la NMO dans la population pédiatrique en Afrique subsaharienne.

Nous avons décrit le cas d’une enfant ayant présenté une atteinte optique bilatérale avant l’atteinte motrice des membres ainsi qu’un syndrome de l’area postrema. Le syndrome de l’area postrema est très évocateur de NMO [4]. Cependant, sa méconnaissance reste source d’erreur de diagnostic : dans notre cas l’origine digestive avait été initialement retenue. Le syndrome de l’area postrema peut précéder une atteinte médullaire et doit donc être identifié et traité de façon précoce [4]. Chez l’enfant, l’atteinte de l’area postrema inaugurale représente 16 % des patients [4]. La série hospitalière décrite par Rania Ben Aounen Tunisie [1] a permis de noter une hétérogénéité clinico-radiologique de la NMO pédiatrique; la névrite optique rétrobulbaire est la forme révélatrice la plus fréquente. Plus récemment, des études comparant des patients pédiatriques et adultes n’ont montré aucune différence dans la manifestation de la maladie en ce qui concerne les caractéristiques cliniques, radiologiques et de laboratoire [8].

Dans notre cas, le grand retard diagnostic explique l’absence de récupération visuelle, car chez notre patiente le diagnostic a été fait à la 4^e^ poussée clinique (retard diagnostic de 19 mois), et l’examen ophtalmologique notait une atrophie optique. Cependant, après les bolus, nous avons noté une franche amélioration clinique, notamment sur le plan moteur. Les corticoïdes sont beaucoup plus accessibles dans nos conditions de travail comparées aux échanges plasmatiques, ayant permis leur utilisation dans notre cas.

Le traitement des poussées est une urgence compte-tenu du risque de séquelles. Il fait essentiellement appel aux corticoïdes administrés par voie intraveineuse à forte dose. En cas d’échec des corticoïdes, les échanges plasmatiques sont plus efficaces que les immunoglobulines polyvalentes par voie veineuse [3]. Les traitements de fond reposent sur les immunosuppresseurs. Le rituximab actuellement utilisé en première ligne reste d’accès difficile du fait de son coût. Une étude de cas rétrospectifs montre que les patients ayant bénéficié de l’escalade thérapeutique par rituximab n’avaient pas présenté de rechute [13]. Chez notre patiente il a été proposé de l’azathioprine en première intention, car beaucoup plus accessible sur le plan financier. En raison du faible nombre et de l’hétérogénéité des études publiées, les données sur la NMO chez l’enfant et l’adolescent sont rares, en particulier en ce qui concerne le traitement et les résultats cliniques [8].

## Conclusion

La NMO pédiatrique est une affection rare, encore largement méconnue dans les pays à ressources limitées. Son diagnostic reste difficile en raison d’une hétérogénéité clinique, du manque d’accessibilité aux examens spécialisés, et d’une faible sensibilisation du corps médical. Dans notre contexte, l’accès aux traitements de fond tels que le rituximab reste une gageure, bien que des dispositifs temporaires de gratuité aient été mis en place depuis avril 2024. Il est essentiel de renforcer le diagnostic précoce, la formation continue des praticiens et de pérenniser l’accès aux traitements innovants afin d’améliorer le pronostic de cette pathologie grave chez l’enfant.

## Remerciements

Tous nos remerciements à Igue Kadidiatou (Médecin pédiatre ayant aidé à la prise en charge) et Offoumou Fiacre Delors (médecin neurologue ayant aidé à la prise en charge).

## Consentement éclairé

Nous avons obtenu le consentement oral de la mère de l’enfant.

## Financement

L’étude n’a bénéficié d’aucun financement.

## Contribution des auteurs et autrices

KADJO Claude Valéry Cédric Aka : conception du rapport de cas, prise en charge diagnostique et thérapeutique du patient, rédaction, révision et validation manuscrit.

YAPO-EHOUNOUD Constance : prise en charge diagnostic et thérapeutique de la patiente, révision et validation manuscrit.

AGBOHOUN Reine Prisca : prise en charge diagnostique et thérapeutique de la patiente rédaction, révision et validation manuscrit.

AKA Arlette Désirée : prise en charge thérapeutique de la patiente, rédaction, révision et validation manuscrit.

## Déclaration de liens d’intérêts

Aucun lien d’intérêt n’a été déclaré.
