# Supplementary material for: Diagnostic and therapeutic challenges of neuromyelitis optica in Côte d’Ivoire: a pediatric case report
Source: Med Trop Sante Int. 2026 Jan 13;6(1):mtsi.v6i1.2026.707. [Article in French] doi: 10.48327/mtsi.v6i1.2026.707 (PMC13180443; doi:10.48327/mtsi.v6i1.2026.707)
Supplement: Related texts

# Research article: Diagnostic and therapeutic challenges of neuromyelitis optica in Côte d'Ivoire: a pediatric case report

## Introduction

Neuromyelitis optica (NMO) is an inflammatory and demyelinating disease of the central nervous system (CNS) that typically progresses in relapses. Although it was long considered a variant of multiple sclerosis (MS), it actually has different pathophysiology, symptomatology, and management than MS [11]. The latest Wingerchuk diagnostic criteria, published in 2015, make diagnosis easier [10]. The discovery of the anti-aquaporin-4 antibody has considerably broadened the range of clinical phenotypes associated with NMO, giving rise to the concept of the “NMO spectrum.” Neuromyelitis Optica Spectrum Disorders (NMOSD) affects women much more frequently than men (7 to 1 in adults; 3 to 1 in children). The average age of onset is around 40 years old, though there are pediatric forms and forms with very late onset (after age 80) [4]. NMO is understudied and underdiagnosed in sub-Saharan Africa, where infectious diseases are major causes of myelopathy. The pediatric form has been studied very little in Africa [6]. Here, we present our first pediatric case and discuss its clinical, diagnostic, therapeutic, and evolutionary characteristics.

## Clinical observation

An 8-year-old Ivorian girl was admitted to the pediatric emergency room on June 15, 2023, with a motor deficit in all four limbs and marked asthenia. The interview revealed an initial episode of decreased binocular visual acuity in November 2021. No etiology was identified, and progression was marked by incomplete improvement in visual acuity. In September 2022, the patient experienced a second episode of severe binocular visual acuity loss with no secondary recovery. The ophthalmological examination data were not specified. A brain MRI performed in September 2022 found no parenchymal lesions.

In May 2023, she was admitted to the emergency department due to uncontrollable vomiting and mild motor deficits in all four limbs. An esophagogastroduodenoscopy was inconclusive in identifying the cause. After receiving treatment for her digestive problems, the vomiting stopped, but the motor deficits persisted.

According to her recent medical history (Fig. 1), three days prior to her admission, she experienced significant asthenia alongside marked facial pruritus, diffuse paresthesia, and a worsening motor deficit. Upon admission, the neurological examination revealed spastic tetraparesis, with motor strength rated at 2/5; diffuse dysesthesia; dysphonia; and binocular blindness with areactive mydriasis. An ophthalmological examination was not performed. The Expanded Disability Status Scale (EDSS) was estimated at 8. Due to the known specificity of autoantibodies and the clinical presentation, as well as the family’s financial constraints, priority was given to biological investigations. An inflammatory disease was suspected and confirmed by the presence of anti-aquaporin-4 (AQP4) antibodies in the serum; anti-myelin oligodendrocyte glycoprotein (MOG) antibodies were negative. Due to the family’s financial limitations, no central nervous system (CNS) imaging was performed during this episode. The patient received a 30-mg/kg/day bolus of corticosteroids for five days, followed by a 1-mg/ kg/day maintenance dose on a tapering schedule for one month. At the end of corticosteroid therapy, the assessment noted an EDSS score of 5.5, as well as an absence of light perception, areflexic semi-mydriasis, and optic atrophy at the fundus from an ophthalmological point of view. Due to difficulties accessing anti-CD20 treatments, azathioprine immunosuppressive therapy was proposed as basic treatment. The course of treatment was marked by a relapse with a worsening motor deficit four months after starting azathioprine. We recommended escalating the treatment by introducing rituximab in April 2024, which was obtained through a free program for AQP4+ NMOSD patients. The patient remained stable on rituximab (500 mg on day 0, 500 mg on day 15, and 1 g every six months) with an EDSS score of 5.

**Figure 1:** Chronological summary of clinical flare-ups and treatment

## Discussion

In Côte d’Ivoire, as in other tropical countries with limited resources, the diagnostic focus remains on infectious diseases such as malaria, tuberculosis, and HIV. This often causes inflammatory demyelinating diseases to be overlooked. These diseases are difficult to diagnose and manage. Currently, rituximab is used as the first-line therapy for NMO, costing approximately 584,348 CFA francs (€890). The cost of treatment remains essentially the responsibility of the family, which is a major limiting factor in our working context. Access to rituximab has been facilitated since April 2024 thanks to a free program established at Cocody University Hospital in Abidjan for AQP4+ NMOSD patients. However, the program’s eligibility criteria, sustainability, and geographic coverage remain uncertain. This situation underscores the necessity of proactive public health policies regarding rare neurological diseases.

NMO is an inflammatory disease of the central nervous system (CNS) that mainly affects the optic nerves and spinal cord [12]. It is more prevalent among individuals of Asian and African descent than those of European descent; however, there is limited data on NMO in Africa [6]. According to data from sub-Saharan Africa, infectious diseases, particularly tuberculosis, dominate the etiologies of myelitis, followed by neoplastic causes [2]. Most studies in sub-Saharan Africa on NMO have noted a delay in diagnosis [6], which could be related to a lack of awareness of this condition. Pediatric NMO is less common than adult NMO. Current data suggest that it accounts for 3-5% of all cases, depending on the diagnostic criteria and whether the AQP4 antibody test is included [9]. The incidence and prevalence of pediatric NMO are not well characterized [7]. To our knowledge, there are no population-based epidemiological data on NMO in children in sub-Saharan Africa. Here, we present our first confirmed case.

A large multicentric study conducted by the Mayo Clinic [5] in pediatric patients with NMO found that the median age at symptom onset was 12 years (range 4 to 18) and that there was a clear female predominance (88%). We present a case of an especially young Ivorian child. Studying a larger number of cases would help determine the average age of NMO onset in the pediatric population in sub-Saharan Africa.

This child presented with bilateral optic involvement before motor involvement of the limbs and area postrema syndrome. Area postrema syndrome is highly suggestive of NMO [4]. However, a lack of awareness of this syndrome can lead to misdiagnosis. In our case, for example, a digestive origin was initially suspected. Area postrema syndrome can precede spinal cord involvement and must therefore be identified and treated early [4]. Among children, initial area postrema involvement accounts for 16% of cases [4]. Rania Ben Aoun’s hospital series in Tunisia [1] noted clinical and radiological heterogeneity in pediatric NMO; retrobulbar optic neuritis is the most common presenting symptom. More recently, studies comparing pediatric and adult patients have shown no difference in disease manifestation in terms of clinical, radiological, and laboratory characteristics [8].

In our case, the long delay in diagnosis explains the lack of visual recovery. Our patient was diagnosed during the fourth clinical flare-up, which occurred 19 months after the initial symptoms appeared. The ophthalmological examination revealed optic atrophy. After the bolus injections, however, we observed clear clinical improvement, particularly in motor function. Corticosteroids are much more accessible than plasma exchanges in our working conditions, which is why we were able to use them in this case.

Given the risk of sequelae, treatment of flare-ups is urgent. It mainly involves high-dose intravenous corticosteroids. If corticosteroids are ineffective, plasma exchange is more effective than intravenous polyvalent immunoglobulins [3]. Background treatments are based on immunosuppressants. Rituximab, which is currently used as the first-line treatment, is difficult to access due to its cost. A retrospective case study shows that patients who received escalating treatment with rituximab did not experience a relapse [13]. For our patient, azathioprine was recommended as the initial treatment because it is much more affordable. Due to the small number of published studies and their heterogeneity, data on NMO in children and adolescents are scarce, particularly with regard to treatment and clinical outcomes [8].

## Conclusion

Pediatric NMO is a rare condition that is still largely unknown in resource-limited countries. Its diagnosis remains difficult due to clinical heterogeneity, lack of access to specialized tests, and low awareness among the medical profession. In our context, access to disease-modifying treatments such as rituximab remains a challenge, although temporary free access schemes have been in place since April 2024. It is essential to strengthen early diagnosis, continuing education for practitioners, and sustainable access to innovative treatments in order to improve the prognosis of this serious disease in children.

## Acknowledgments

Our thanks go to Igue Kadidiatou (pediatrician who helped with treatment) and Offoumou Fiacre Delors (neurologist who helped with treatment).

## Informed consent

We obtained verbal consent from the child’s mother.

## Funding

The study did not receive funding.

## Authors’ contribution

KADJO Claude Valéry Cédric Aka: case report design, diagnostic and therapeutic management of the patient, writing, revision, and manuscript validation.

YAPO-EHOUNOUD Constance: diagnostic and therapeutic management of the patient, manuscript revision and validation.

AGBOHOUN Reine Prisca: diagnostic and therapeutic management of the patient, manuscript writing, revision, and validation.

AKA Arlette Désirée: therapeutic management of the patient, writing, revision, and validation of the manuscript.

## Conflicts of interest

The authors declare no conflicts of interest
